# Through-Wall Image Enhancement Using Fuzzy and QR Decomposition

**DOI:** 10.1155/2014/487506

**Published:** 2014-02-23

**Authors:** Muhammad Mohsin Riaz, Abdul Ghafoor

**Affiliations:** ^1^Centre for Advanced Studies in Telecommunication (CAST), Comsats, Islamabad, Pakistan; ^2^Department of Electrical Engineering, College of Signals, National University of Sciences and Technology (NUST), Islamabad, Pakistan

## Abstract

QR decomposition and fuzzy logic based scheme is proposed for through-wall image enhancement. QR decomposition is less complex compared to singular value decomposition. Fuzzy inference engine assigns weights to different overlapping subspaces. Quantitative measures and visual inspection are used to analyze existing and proposed techniques.

## 1. Introduction

Mapping of scenes behind obstacles (including building wall, rubbers, grass, etc.) using through-wall imaging (TWI) is an unfolded research domain. Different military and commercial applications (including antiterrorism, hostage rescue and surveillance, etc. [1]) can benefit from TWI. Beside other challenges, minimization of unwanted artifacts (clutters/noise) has enjoyed special importance over last few years [2–13]. These unwanted artifacts significantly decrease target detection and recognition capabilities.

Existing TWI image enhancement (clutter removal) techniques include background scene subtraction (only feasible if with and without target images are available) [2], spatial filtering (assuming wall homogeneity at low frequencies) [3], wall modeling and subtraction (requiring complex process for inhomogeneous walls) [4, 5] Doppler filtering (applicable for moving targets only) [6], image fusion (requiring multiple data of the same scene) [7], and statistical techniques [8–13].

In this paper, a TWI image enhancement (clutter reduction) technique using QR decomposition (QRD) and fuzzy logic is presented (preliminary results presented in [13]). Weights are assigned to different QRD subspaces using fuzzy inference engine. Simulation results evaluated using mean square error (MSE), peak signal to noise ratio (PSNR), improvement factor (IF), and visual inspection (based on miss detection (MD) and false detection (FD)) are used to verify the proposed scheme.

## 2. Proposed Image Enhancement Using QRD

Let the input image *M* (having dimensions *G* × *H*) be decomposed into different subspaces (*M*_cl⁡_, *M*_tar_, and *M*_no_) using singular value decomposition (SVD) as

(1) $$\begin{array}{c} M=\underset{M_{\mathrm{cl}}}{\underset{︸}{\sum_{g=1}^{l_{1}}s_{g}u_{g}v_{g}^{T}}}+\underset{M_{\text{tar}}}{\underset{︸}{\sum_{g=l_{1}+1}^{l_{2}}s_{g}u_{g}v_{g}^{T}}}+\underset{M_{\text{no}}}{\underset{︸}{\sum_{g=l_{2}+1}^{G}s_{g}u_{g}v_{g}^{T}}}, \end{array}$$

where *U* and *V* are singular vector matrices and *S* contains singular values. As discussed in [13], conventional SVD for TWI image enhancement assumes that the target is limited to the second spectral component only; that is,

(2) $$\begin{array}{c} M_{\text{SVD}}=s_{2}u_{2}v_{2}^{T}. \end{array}$$

Besides the high computational complexity of SVD which is 4*G* ^2^ *H* + 8*GH* ^2^ + 9*H* ^3^ [14], the statement of target containment in the second spectral component is not always true. To cater the above issues, QRD and fuzzy logic based scheme is proposed. The image *M* can be decomposed into an orthogonal unitary matrix *Q* (having dimensions *G* × *H* and column vectors *q*_*g*_) and an upper triangular matrix *R* (having dimensions *G* × *H* and row vectors *r*_*g*_), that is,

(3) $$\begin{array}{c} M=QR. \end{array}$$

Table 1 shows the accuracy, stability, and complexity analysis of different QRD algorithms (classical and modified Gram-Schmidt (CGS, MGS), Givens decomposition, Householder transformation (HT), etc. [14]) for TWI.

Identical to SVD, the first subspace *M*_1_ = *q*_1_ *r*_1_ represents wall clutters and rest subspaces contain targets and noise. Note that due to overlapping boundaries of targets and noise, it is difficult to extract target subspaces accurately. Foregoing in view, a weighting QRD based scheme is proposed to enhance targets. The enhanced image *M*_tar_ is

(4) $$\begin{array}{c} M_{\text{tar}}=\sum_{g=2}^{G}w_{g}q_{g}r_{g}, \end{array}$$

where *w*_*g*_ are weights applied to different subspaces. Fuzzy logic is used for the automatic weight assignment [15].

### 2.1. Input and Output MFs

Let *ξ*_*g*_ = ||*r*_*g*_|| and Δ*ξ*_*g*_ = ||*r*_*g*_|| − ||*r*_*g*+1_|| be norms and norm differences, respectively. Note that high value of *ξ*_*g*_ and Δ*ξ*_*g*_ the corresponding subspace *q*_*g*_ *r*_*g*_ more likely contains target(s) and is therefore enhanced by applying heavy weights (and vise versa).

Three Gaussian membership functions (MFs) $\zeta_{X^{x}}(x_{1})=\exp\left(-{\left(\left(c_{1}-{\overset{-}{c}}_{1}^{\left(x\right)}\right)/\sigma_{1}^{\left(x\right)}\right)}^{2}\right)$ and (*x* ∈ {High, Medium, Low}) are defined for *ξ*_*g*_. Similarly $\zeta_{Y^{y}}(x_{2})=\exp\left(-{\left(\left(c_{2}-{\overset{-}{c}}_{2}^{\left(y\right)}\right)/\sigma_{2}^{\left(y\right)}\right)}^{2}\right)$ and (*y* ∈ {High, Medium, Low}) are defined for Δ*ξ*_*g*_, where {*c*_1_, *c*_2_}∈[0,1], ${\overset{-}{c}}_{1}^{\left(x\right)}$, ${\overset{-}{c}}_{2}^{\left(y\right)}$ and *σ*_1_^(*x*)^, *σ*_2_^(*y*)^ are means and variances of fuzzy sets, respectively.

*K*-means algorithm [16] is used to adjust the fuzzy parameters. *ξ*_*k*_ and Δ*ξ*_*h*_ are first clustered into three groups based on respective histograms. The means and variances, respectively, of each group are used as centers ${\overset{-}{c}}_{1}^{\left(x\right)}$, ${\overset{-}{c}}_{2}^{\left(y\right)}$ and spreads *σ*_1_^(*x*)^, *σ*_2_^(*y*)^ of MFs. Five equally spaced output MFs $\zeta_{Z^{z}}\left(d\right)=-{\left(\left(d-{\overset{-}{d}}^{\left(z\right)}\right)/\varrho^{\left(z\right)}\right)}^{2}\;\;(z\in \{\text{Very}\;\;\text{High},\text{High},\text{Medium},\text{Low},\text{Very}\;\;\text{Low}\}$), where mean ${\overset{-}{d}}^{\left(z\right)}$ and variance *ϱ* ^(*z*)^ are used.

### 2.2. Product Inference Engine (PIE)

Gaussian fuzzifier maps the input *ξ*_*g*_ and Δ*ξ*_*g*_ as

(5) $$\begin{array}{c} \zeta_{XY}\left(c_{1},c_{2}\right)=\exp\left\{-{\left(\frac{c_{1}-\xi_{g}}{v_{1}}\right)}^{2}\right\}\exp\left\{-{\left(\frac{c_{2}-\Delta \xi_{h}}{v_{2}}\right)}^{2}\right\}, \end{array}$$

where *v*_1_ and *v*_2_ are parameters used for input noise suppression and are chosen as *v*_1_ = 2max⁡_*x*_ *σ*_1_^(*x*)^ and *v*_2_ = 2max⁡_*y*_^3^ *σ*_2_^(*y*)^ [15].

Fuzzy IF-THEN rules for image enhancement are the following.

 Rule 1: IF *ξ*_*h*_ is *X* ^High^ and Δ*ξ*_*h*_ is *Y* ^High^, THEN *w*_*h*_^PIE^ is *Z* ^Very  High^.
 Rule 2: IF *ξ*_*h*_ is *X* ^Med^ and Δ*ξ*_*h*_ is *Y* ^High^, THEN *w*_*h*_^PIE^ is *Z* ^High^.
 Rule 3: IF *ξ*_*h*_ is *X* ^High^ and Δ*ξ*_*h*_ is *Y* ^Med^, THEN *w*_*h*_^PIE^ is *Z* ^High^.
 Rule 4: IF *ξ*_*h*_ is *X* ^Med^ and Δ*ξ*_*h*_ is *Y* ^Med^, THEN *w*_*h*_^PIE^ is *Z* ^Med^.
 Rule 5: IF *ξ*_*h*_ is *X* ^High^ and Δ*ξ*_*h*_ is *Y* ^Low^, THEN *w*_*h*_^PIE^ is *Z* ^Med^.
 Rule 6: IF *ξ*_*h*_ is *X* ^Low^ and Δ*ξ*_*h*_ is *Y* ^Med^, THEN *w*_*h*_^PIE^ is *Z* ^Med^.
 Rule 7: IF *ξ*_*h*_ is *X* ^Med^ and Δ*ξ*_*h*_ is *Y* ^Low^, THEN *w*_*h*_^PIE^ is *Z* ^Low^.
 Rule 8: IF *ξ*_*h*_ is *X* ^Low^ and Δ*ξ*_*h*_ is *Y* ^High^, THEN *w*_*h*_^PIE^ is *Z* ^Low^.
 Rule 9: IF *ξ*_*h*_ is *X* ^Low^ and Δ*ξ*_*h*_ is *Y* ^Low^, THEN *w*_*h*_^PIE^ is *Z* ^Very  Low^.

The output of PIE using individual rule based inference, Mamdani implication, algebraic product for *t*-norm, and max operator for *s*-norm [15] is

(6) $$\begin{array}{c} \zeta_{Z^{\prime }}\left(d_{g}\right)=\underset{\left\{x,y,z\right\}}{\max}\left[\underset{\left\{c_{1},c_{2}\right\}}{\sup}\zeta_{XY}\left(c_{1},c_{2}\right)\zeta_{X^{x}}\left(c_{1}\right)\zeta_{Y^{y}}\left(c_{2}\right)\zeta_{Z^{z}}\left(d_{g}\right)\right]. \end{array}$$

The weights *w*_*g*_^PIE^ are then computed as

(7) $$\begin{array}{c} w_{g}^{\text{PIE}}=\frac{\sum_{z}{\overset{-}{d}}^{\left(z\right)}\varpi_{g}^{\left(z\right)}}{\sum_{z}=1\varpi_{h}^{\left(z\right)}}, \end{array}$$

where *ϖ*_*g*_^(*z*)^ is the height of *ζ*_*Z*′_(*d*_*g*_) in output MFs [15].

### 2.3. Takagi-Sugeno (TS) Inference

In contrast to PIE, TS inference engine adjusts the output MFs using adaptive and/or optimization techniques [17]. The TS rule-base (IF-THEN) for computing weights *w*_*g*_^TS^ is

(8) $$\begin{array}{c} \text{IF}\;\xi_{g}\;\text{is}\;X^{j_{1}}\;\text{AND}\;\Delta \xi_{g}\;\text{is}\;Y^{j_{2}}\\ \text{THEN}\;p^{\left(j_{1}+j_{2}-1\right)}={\left(\frac{1}{1+\exp\left\{-\xi_{g}\right\}+\exp\left\{-\Delta \xi_{g}\right\}}\right)}^{j_{1}+j_{2}-1}. \end{array}$$

Note that the output reduces for large *j*_1_ + *j*_2_ (which is desirable). The aggregated weights *w*_*g*_^TS^ are

(9) $$\begin{array}{c} w_{g}^{\text{TS}}=\frac{\sum_{j_{1}=1}^{3}\sum_{j_{2}=1}^{3}p^{\left(j_{1}+j_{2}-1\right)}t\left\{\zeta_{X^{j_{1}}}\left(\xi_{k}\right),\zeta_{Y^{j_{2}}}\left(\Delta \xi_{g}\right)\right\}}{\sum_{j_{1}=1}^{3}\sum_{j_{2}=1}^{3}t\left\{\zeta_{X^{j_{1}}}\left(\xi_{g}\right),\zeta_{Y^{j_{2}}}\left(\Delta \xi_{g}\right)\right\}}, \end{array}$$

where *t* represents algebraic product (intersection operator).

## 3. Simulation and Results

Experimental setup for TWI is constructed using Agilent's vector network analyzer (VNA) which generates stepped frequency waveforms between 2 GHz and 3 GHz (1 GHz band width (BW)) having step size of Δ*f* = 5 MHz and step size *N*_*f*_ = 201. Maximum range is *R*_max⁡_ = 30 m and range resolution is Δ*R* = 0.15 m.

Broadband horn antenna which is mounted on two-dimensional scanning frame (having dimensions 2.4 m × 3 m (width × height) and can slide along cross range and height) operates in monostatic mode with 12 dB gain. Thickness of the wall is 5 cm and relative permittivity and permeability are 2.3 and 1, respectively. The frame is placed 0.03 m away from wall and scanning is controlled by a microcontroller based mechanism. The scattering parameters are recorded at each step and transferred to a local computer for image reconstruction and processing. Received data is converted into time domain and beamforming algorithm is used for image reconstruction. Existing and proposed image enhancement algorithms are simulated in MATLAB and quantitative analysis is performed using MSE, PSNR, IF, FD, MD, and visual inspection:

(10) $$\begin{array}{c} \text{MSE}=\frac{1}{G\times H}\sum_{g=1}^{G}\sum_{h=1}^{H}{\left(M_{bs}(g,h)-M_{\text{tar}}(g,h)\right)}^{2},\\ \text{PSNR}\left(\text{dB}\right)=10\;\log_{10}\frac{1}{\text{MSE}},\\ \text{IF}\left(\text{dB}\right)=10{\;\log}_{10}\left[\frac{P_{M_{\text{tar}},t}\times P_{M,c}}{P_{M,t}\times P_{M_{\text{tar}},c}}\right], \end{array}$$

where *M*_*bs*_ is a reference image obtained by the difference of image, with and without target. *P*_*M*_tar_,*t*_ and *P*_*M*_tar_,*c*_ are average pixel values of target and clutter in enhanced image, respectively. *P*_*M*,*t*_ and *P*_*M*,*c*_ are average pixel values of target and clutter in the original image, respectively.

MD is defined as “target was present in the original image, but was not detected in enhanced image.” FD is defined as “target was not present in the original image, but was detected in enhanced image.” For calculating FD and MD, a threshold is calculated using global thresholding algorithm [18].

**Figure 1:**
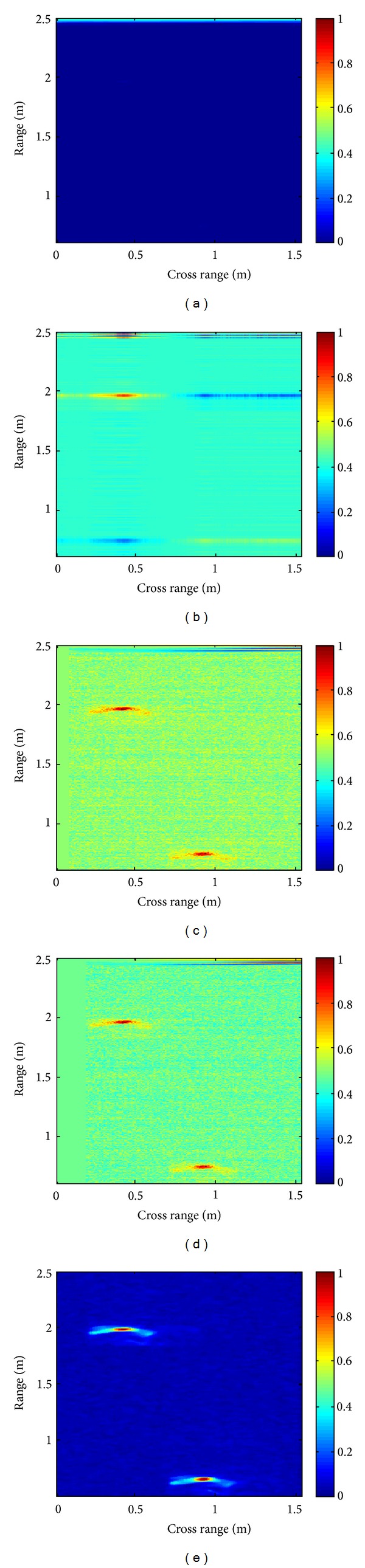
Image with two targets. (a) Original image. (b) SVD [11]. (c) Proposed fuzzy QRD (PIE). (d) Proposed fuzzy QRD (TS). (e) Background subtracted reference image.

Figure 1 shows the original B-scan containing two targets, the background subtracted reference and enhanced images, using existing SVD and proposed QRD based schemes. It can be observed that proposed schemes detect both targets whereas SVD based scheme is unable to locate both targets accurately.

**Figure 2:**
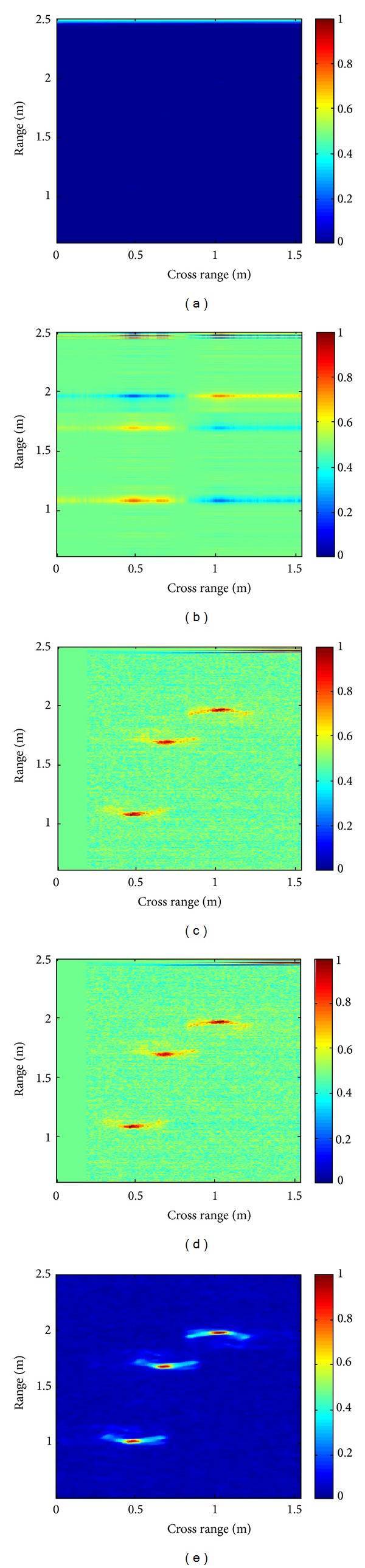
Image with three targets. (a) Original image. (b) SVD [11]. (c) Proposed fuzzy QRD (PIE). (d) Proposed fuzzy QRD (TS). (e) Background subtracted reference image.

Figure 2 shows another example containing three targets. The proposed scheme detects all targets and provides a better target to background ratio compared to SVD based scheme. It is further noted that the proposed TS inference based scheme provides better results compared to PIE.

Table 2 shows that proposed fuzzy QRD schemes are better (as compared to the SVD image enhancement scheme) in terms of MSE, PSNR, IF, MD, and FD.

## 4. Conclusion

QRD and fuzzy logic based image enhancement scheme is proposed for TWI. Compared with SVD, QRD provides less computational complexity. PIE and TS inference engines are used to assign weights to different QRD subspaces. Simulation results compared on visual and quantitative analysis show the significance of the proposed scheme.

## Figures and Tables

**Table 1:** Comparison analysis of QRD algorithms for TWI.

Algorithms	Accuracy				Complexity	Stability
	*M* − *QR*	*Q* ^*T*^ *Q* − *I*	*Q* ^*T*^ *M* − *R*	*MR* ^−1^ − *Q*		
CGS QR	1.091 × 10^−16^	1.310 × 10^−10^	6.724 × 10^−14^	9.913 × 10^−14^	2*GH* ^2^	Unstable
MGS QR	1.075 × 10^−16^	4.922 × 10^−13^	4.842 × 10^−14^	1.083 × 10^−12^	2*GH* ^2^	Stable
HT QR	1.291 × 10^−15^	3.795 × 10^−15^	3.333 × 10^−16^	1.263 × 10^−12^	$4G^{2}H+2GH^{2}+\frac{2}{3}H^{3}$	Stable
Givens QR	7.532 × 10^−16^	6.702 × 10^−15^	2.711 × 10^−16^	1.118 × 10^−12^	$8G^{2}H+2GH^{2}+\frac{2}{3}H^{3}$	Stable

**Table 2:** MSE, PSNR, IF, MD, and FD comparison.

Scenario	Scheme	MSE	PSNR	IF	MD	FD
Two targets	SVD [11]	0.2726	5.6442	8.1258	1	0
	Fuzzy QRD (PIE)	0.1970	7.0553	11.2587	0	0
	Fuzzy QRD (TS)	0.1726	7.6296	11.5870	0	0

Three targets	SVD [11]	0.2814	5.5068	7.1265	1	1
	Fuzzy QRD (PIE)	0.1933	7.1377	10.8715	0	0
	Fuzzy QRD (TS)	0.1824	7.3898	11.0127	0	0
